# TARGETING P21CIP1-HIGHLY-EXPRESSING SENESCENT CELLS TO IMPROVE HEALTHSPAN IN PROGEROID MICE

**DOI:** 10.1093/geroni/igad104.3618

**Published:** 2023-12-21

**Authors:** Binsheng Wang, Lichao Wang, Ming Xu

**Affiliations:** UConn Health, Farmington, Connecticut, United States; UConn Health, Farmington, Connecticut, United States; UConn Health, Farmington, Connecticut, United States

## Abstract

Aging is the leading risk factor for a variety of chronic diseases. Emerging evidence suggests that modulating fundamental aging processes such as cellular senescence may delay the onset of most chronic conditions as a group and increase healthy lifespan. Cellular senescence refers to the essentially irreversible growth arrest that occurs when cells experience stress. One common feature of senescent cells is the high expression level of p16Ink4a and/or p21Cip1. We and others have demonstrated the causal roles of p16Ink4a-highly-expressing (p16high) senescent cells in various age-related conditions. However, the role of p21high senescent cells in aging remains largely unknown. To address these questions, we have generated and validated a novel p21-Cre mouse model containing p21 promoter driving inducible Cre. By crossing with floxed mice, we managed to monitor, sort, image, eliminate, or modulate p21high cells in vivo. We investigated the maximal walking speed and grip strength of klotho (kl)-/- progeroid mice weekly starting from 5 weeks old until death. We showed that clearance of p21high cells can improve physical function in kl-/- mice for a long time period. Additionally, inactivation of NF-κB specifically in p21high cells also can improve tissue function in kl-/- mice. This study will provide invaluable research tools and pinpoint the role of cellular senescence in age-related tissue dysfunction.

